# Effects of blocking mGluR5 on primate dorsolateral prefrontal cortical neuronal firing and working memory performance

**DOI:** 10.1007/s00213-020-05661-2

**Published:** 2020-09-16

**Authors:** Sheng-Tao Yang, Min Wang, Veronica Galvin, Yang Yang, Amy F. T. Arnsten

**Affiliations:** Department of Neuroscience, Yale University School of Medicine, New Haven, CT 06510 USA

**Keywords:** mGluR5, Prefrontal cortex, Working memory, Glutamate, Rhesus monkey, Physiology, Cognition

## Abstract

**Rationale:**

Metabotropic glutamate type 5 receptor (mGluR5) antagonists are under development for treating cognitive disorders such as Fragile X syndrome and Alzheimer’s disease, largely based on success in mouse models, where post-synaptic mGluR5 stimulation weakens synaptic functions in hippocampus. However, human trials of mGluR5 antagonists have yet to be successful. This may be due in part to the differing effects of mGluR5 in hippocampus vs. prefrontal cortex, as mGluR5 are primarily post-synaptic in rodent hippocampus, but are both pre- and post-synaptic in the dorsolateral prefrontal cortical (dlPFC) circuits known to subserve working memory.

**Objectives and methods:**

The current study examined the effects of the selective mGluR5 negative allosteric modulator, MTEP (3-((2-Methyl-1,3-thiazol-4-yl)ethynyl)pyridine hydrochloride), on neuronal firing and working memory performance in aging rhesus monkeys with naturally occurring impairments in neuronal firing and cognitive performance.

**Results:**

We found that iontophoresis of MTEP directly onto dlPFC “Delay cells” had an inverted U dose-response, where low doses tended to enhance task-related firing, but higher doses suppressed neuronal firing. Similar effects were seen on cognitive performance following systemic MTEP administration (0.0001–0.1 mg/kg), with MTEP producing erratic dose-response curves. In the subset of monkeys (50%) that showed replicable improvement with MTEP, co-administration with the mGluR5 PAM, CDPPB (3-Cyano-*N*-(1,3-diphenyl-1*H*-pyrazol-5-yl)benzamide), blocked MTEP beneficial effects, consistent with mGluR5 actions.

**Conclusions:**

The mixed effects of MTEP on cognitive performance may arise from opposing actions at pre- vs. post-synaptic mGluR5 in dlPFC. These data from monkeys suggest that future clinical trials should include low doses, and identification of potential subgroup responders.

## Introduction

The metabotropic glutamate receptor type 5 (mGluR5) is a potential therapeutic target for cognitive disorders, largely based on encouraging results from mouse models of human ailments. mGluR5 are part of the group 1 family of metabotropic glutamate receptors, which couple through Gq signaling (Niswender and Conn 2010). Activation of mGluR5/Gq signaling generates IP3 and DAG, increasing IP3-mediated calcium release from the smooth endoplasmic reticulum (SER) and activating protein kinase C (PKC) (Kawabata et al. 1996). Research suggests that mGluR5 may be involved in pathological actions in cognitive disorders, and thus blockade of these receptors may be therapeutic. Much of this work has focused on mGluR5 actions in mouse hippocampal neurons, where mGluR5 are primarily post-synaptic (Shigemoto et al. 1997), and can induce long-term depression (LTD) at NMDAR synapses (O’Riordan et al. 2018).

Mouse models of autism spectrum disorders, which are typified by deficits in social cognition, were the first to suggest that blocking mGluR5 may be therapeutic (Krueger and Bear 2011). This work has focused on Fragile X syndrome, where a trinucleotide repeat (CGG) expansion mutation in FMR1 causes reduction or loss of expression of FMRP, which normally serves to repress mGluR5-activated mRNA translation. Thus, this mutation leads to increased mGluR5 expression and increased LTD in mouse hippocampus (Bear et al. 2004). While mGluR5 antagonists were successful in normalizing function in mouse models, so far these agents have failed in human clinical trials (Berry-Kravis et al. 2018). However, the clinical trials had several limitations, e.g., the patients were all over 12 years old, drug treatment was only for 3 months, and there were large placebo effects (Berry-Kravis et al. 2018). Importantly, most studies did not measure social cognition, and the trials were not designed to detect whether a subgroup responded to drug, which is often the case for mental disorders.

Research also suggests that mGluR5 may be involved in exacerbating the degenerative process in Alzheimer’s disease (AD). Early studies found that activation of post-synaptic mGluR5 increases amyloid precursor protein (APP) and beta amyloid (Aβ) expression via release from FMRP repression, an extension of the research on Fragile X syndrome (Westmark and Malter 2007). Subsequent studies showed that soluble Aβ peptides can stimulate mGluR5 to induce LTD (Hu et al. 2014), and drive abnormal calcium signaling, causing synaptic deterioration and aggravating the degenerative process (Renner et al. 2010). Studies of both mouse models and human AD post-mortem tissue further revealed that Aβ oligomers interact with mGluR5 via prion protein (PrP), which activates fyn kinase to induce tau phosphorylation, NMDAR retraction from the PSD, and spine loss (Um et al. 2013). Taken together, these data suggested that blockade of mGluR5 may be protective against AD.

**Fig. 1:**
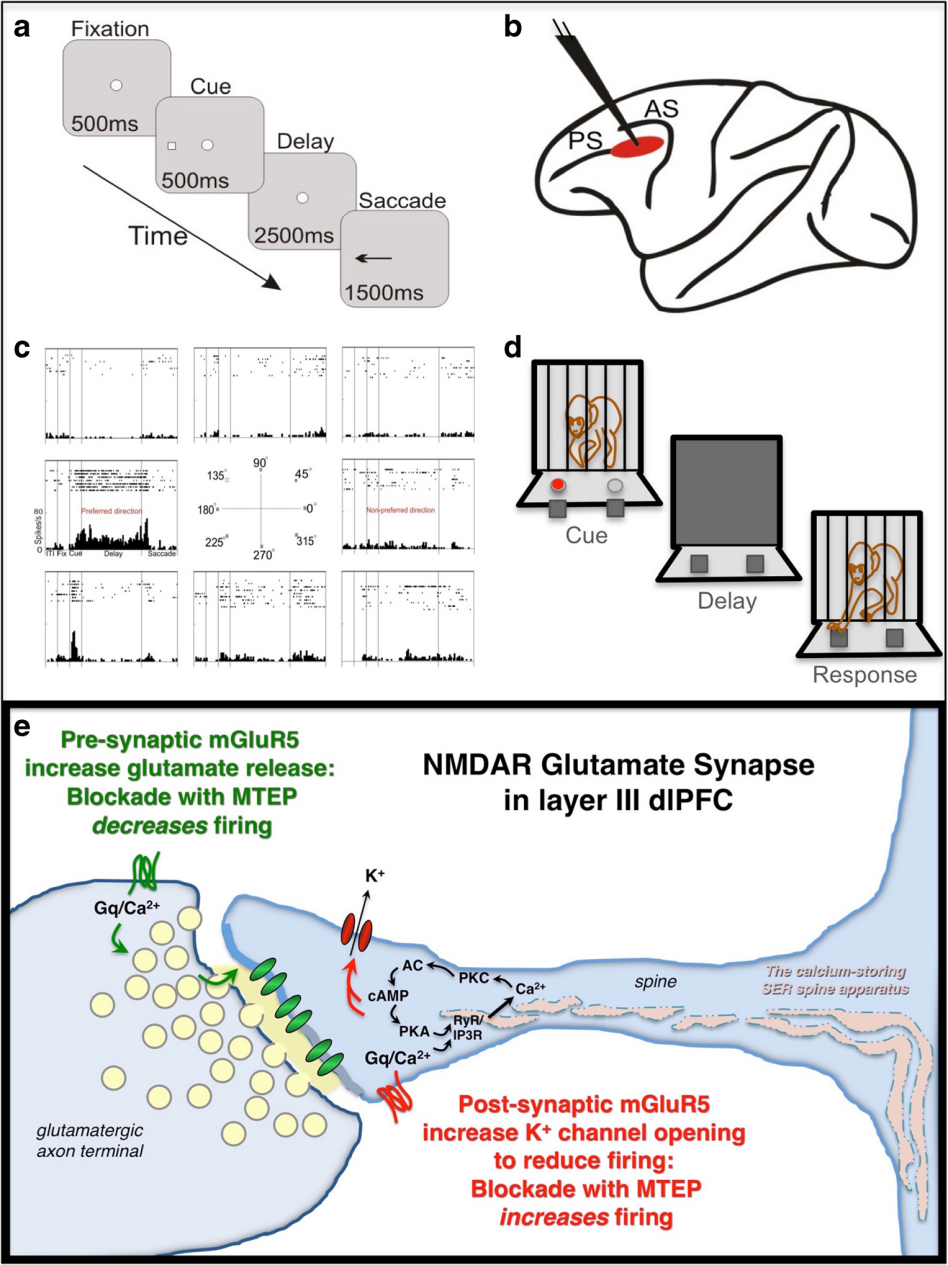
The experimental paradigm. **a** The spatial working memory Oculomotor Delayed Response task used for physiological experiments, where a monkey makes an eye movement to the remembered location. **b** The site of neuronal recordings in dlPFC. PS, principal sulcus; AS, arcuate sulcus. **c** A representative Delay cell from dlPFC with persistent firing across the delay epoch for its preferred direction (180°), but not other, non-preferred locations. **d** The traditional, manual version of the spatial delayed response task. **e** Schematic illustration of potential mGluR5 actions based on their pre- and post-synaptic immunoEM localization in rhesus monkey layer III dlPFC. See text for details

Although research to date has primarily focused on the rodent hippocampus, translation to humans requires understanding of molecular actions in the association cortices, which greatly expand in primates and govern higher cognition, yet are often governed differently at the molecular level than the hippocampus. For example, the neuronal circuits in the dorsolateral prefrontal cortex (dlPFC) that generate working memory and abstract reasoning are modulated differently than hippocampal circuits (Arnsten et al. 2012). Neurons in the primate dlPFC have the ability to maintain spatially tuned firing across the delay epoch during a spatial working memory task, providing the cellular basis for working memory (Fuster and Alexander 1971; Goldman-Rakic 1995). These specialized neurons are termed “Delay cells” (Fig. 1). The precisely timed and tuned pattern of Delay cell firing is generated by microcircuits in dlPFC deep layer III, which have extensive recurrent excitation to maintain persistent firing, and lateral inhibition from GABA interneurons to refine spatial tuning (Goldman-Rakic 1995; González-Burgos et al. 2000; González-Burgos et al. 2005). The persistent firing across the delay period depends on NMDAR stimulation, including those with NR2B subunits that are found exclusively in the post-synaptic density (Wang et al. 2013). As summarized in Fig. 1e, layer III spines contain the molecular machinery for feedforward, cAMP-calcium signaling to reduce neuronal firing by opening nearby K^+^ channels, thus providing negative feedback in a recurrent excitatory circuit (Arnsten et al. 2012). These actions may be engaged by post-synaptic mGluR5 in layer III dlPFC (Muly et al. 2003), playing a parallel role to the LTD described in hippocampus. Importantly, feedforward calcium-cAMP-K^+^ signaling is dysregulated in the aging dlPFC, leading to reduced Delay cell firing and impaired working memory that begins in middle age (Wang et al. 2011). Thus, one could posit that mGluR5 blockade may be particularly helpful in the aging primate cortex.

However, in contrast to rodent hippocampus where mGluR5 are primarily post-synaptic (Shigemoto et al. 1997), immunoEM of primate layer III dlPFC has shown that mGluR5 are pre-synaptic as well as post-synaptic in the newly evolved dlPFC circuits (Muly et al. 2003). Studies in rodents have shown that stimulation of pre-synaptic mGluR5 can increase glutamate release in mPFC (Isherwood et al. 2018), and spinal cord (Park et al. 2004). Similar pre-synaptic actions in primate dlPFC would likely increase glutamate release onto NMDAR and increase delay-related neuronal firing. Conversely, blockade of pre-synaptic mGluR5 in dlPFC might reduce glutamate release and decrease working memory–related neuronal firing. Thus, a concern for a potential therapeutic would be that opposing actions at pre- vs. post-synaptic mGluR5 in dlPFC may counteract each other and erode beneficial actions.

The current study examined the effects of the selective mGluR5 negative allosterioc modulator (NAM), MTEP (3-((2-Methyl-1,3-thiazol-4-yl)ethynyl)pyridine hydrochloride) (Lea and Faden 2006), on dlPFC Delay cell firing and working memory performance in aging monkeys. Results showed that MTEP produced erratic and narrow inverted U dose/response curves for both neuronal firing and cognitive performance, consistent with competing actions at pre- vs. post-synaptic receptors, but with replicable improvement in a subgroup of animals.

## Materials and methods

All research was approved by the Yale IACUC and was in accordance with NIH guidelines.

The research investigated the effects of the mGluR5 NAM, MTEP, at the cellular and behavior levels, testing how (1) local administration onto dlPFC Delay cells effected neuronal firing as monkeys performed a spatial WM task and (2) systemic administration of MTEP effected behavioral performance of a spatial WM task.

### Physiological recordings

#### Subjects

Two aging rhesus (*Macaca mulatta*) monkeys (a 15-year-old male, and a 21-year-old female who was 29 years at the time of subsequent behavioral testing) were used in the current study, and cared for under the guidelines of the National Institutes of Health and the Yale IACUC.

#### Oculomotor delayed response task

The monkeys were seated in primate chairs with their heads fixed, and faced a 27-inch computer monitor 30 inches in front of them. The monkeys’ eye positions were monitored with the ISCAN Eye Movement Monitoring System (ISCAN, Burlington, MA). The monkeys were trained in the visuo-spatial oculomotor delayed response (ODR) task (Fig. 1a), which required the subject to make a memory-guided saccade to a remembered visuo-spatial target for juice reward. The position of the stimulus was randomized over trials such that it had to be remembered on a trial-by-trial basis. The inter-trial intervals (ITI) were at least 3 s. Monkeys performed 1000–1500 trials per session. Patients with schizophrenia have been shown to be impaired on a human version of this task (Keedy et al. 2006).

#### In vivo single unit recordings and iontophoresis

MRI-guided placement of chronic recording chambers allowed single unit recording from the caudal principal sulcal dlPFC (Fig. 1b). MTEP (Tocris, Bio-Techne Corp, Minneapolis, MN) was dissolved at 0.01M concentration in sterile water with pH 3–4. Iontophoretic electrodes were constructed with a 20-μm-pitch carbon fiber (ELSI, San Diego, CA) inserted in the central barrel of a seven-barrel non-filamented capillary glass (Friedrich and Dimmock, Millville, NJ). The assembly was pulled using a multipipette electrode puller (PMP-107L, Microdata Instrument Inc., South Plainfield, NJ) and the tip was beveled to obtain the finished electrode. Finished electrodes had impedances of 0.3–1.5 MΩ (at 1 kHz) and tip sizes of 30–40 μm. The outer barrels of the electrode were then filled with drug solutions (two consecutive barrels each) and the solutions were pushed to the tip of the electrode using compressed air. A Neurophore BH2 iontophoretic system (Medical Systems Corp., Greenvale, NY) was used to control the delivery of the drug. Retaining currents of – 5 to – 10nA were used in a cycled manner (1 s on, 1 s off) when not applying drugs. MTEP was applied at ejection currents from 5 to 40nA. Please note that parallel studies with the selective mGluR5 PAM, CDPPB, could not be performed, as CDPPB does not have an electric charge necessary for iontophoretic application. Drug ejection did not create noise in the recording, and there was no systematic change in either spike amplitude or time course at any ejection current. Further recording details can be found in Wang et al. (2007). We classified four different kinds of ODR task–related cells: Fixation cells, Cue cells, Delay cells, and Response cells. A typical Delay cell is shown in Fig. 1c, where the neuron is able to maintain firing across the delay epoch for its preferred direction, e.g., 180°.

Neuronal activities were first collected from the cell under a control condition in which at least eight trials at each of 8 cue locations were obtained. Upon establishing the stability of the cells’ activity, this control condition was followed by iontophoretic application of drug. Dose-dependent effects of the drug were tested in two or more consecutive conditions, which then was followed by a Recovery condition. Drug was continuously applied at a relevant current throughout a given condition. Each condition had ~ 8 (6–12) trials at each location to allow for statistical analyses of drug effects.

#### Data analyses

Each trial was divided into four epochs – initial fixation, cue, delay, and response (Saccade). Data analysis was performed in MATLAB, SPSS, and GraphPad Prism 7. Spike density functions were constructed in 50ms windows. One-way ANOVA or two-way ANOVA assessed the effects of drug application on task-related activity.

### Behavioral assessments of working memory performance

#### Subjects

Ten adult and aged rhesus monkeys (*Macaca mulatta*, 8 females and 2 males, ages 12–32 years,) were pair-housed under standard laboratory conditions with individualized environmental enrichment. Monkeys were tested for highly palatable rewards (e.g., raisins, chocolate chips) to minimize the need for dietary regulation. Animals were fed monkey chow (Purina Mills, St. Louis, MO) as well as fresh fruit and vegetables immediately following testing; water was available ad libitum. One aged female monkey (AR) had previously participated in the physiological recordings prior to the systemic behavioral analyses, and thus provided the rare opportunity to compare neuronal and behavioral results.

#### Manual spatial working memory task

The monkeys had been previously trained on a manual, multiple delay version of the delayed response spatial working memory task in a Wisconsin General Testing Apparatus (Fig. 1d; see Arnsten et al. (1988) for details). All monkeys performed near perfectly at 0 s, where the opaque screen is not lowered, and exhibit increasing errors with longer delays. The delay lengths were adjusted for each animal so that they had a stable baseline performance of 67–80% correct, leaving room for improvement of impairment in performance with drug treatment. Animals were tested by experimenters who were highly familiar with the normative behavior of each monkey, but blind to drug treatment conditions.

#### Behavioral ratings

Sedation/agitation and aggression during cognitive testing were assessed using 9-point rating scales: The sedation/agitation scale ranged from IV: too sedated to test to –IV: too agitated to test. The aggression scale ranged from IV: much less aggressive than usual to –IV: too aggressive to test safely.

#### Drug administration

Monkeys were injected (i.m.) with sterile saline vehicle or MTEP (Lea and Faden 2006) 60 min before cognitive testing. A wide range of doses (0, 0001, 0.001, 0.01, and 0.1 mg/kg) were tested; pilot experiments with a higher dose (1.0 mg/kg) showed no evidence of improvement. Monkeys were required to exhibit stable baseline performance for 2 consecutive test sessions prior to subsequent drug treatment, with a minimum washout period of at least 10 days. MTEP dosages that improved performance above baseline levels were repeated to test for replication. Two of the oldest monkeys (27, 32 years) died before their dose/response curves could be completed; thus, the 0.0001 mg/kg dose contained *n* = 8 and the 0.1 mg/kg dose *n* = 9.

Following the MTEP characterization, the subset of aged monkeys (*n* = 5, 4 female and 1 male) that showed replicable improvement with MTEP was challenged with the mGluR5 positive allosteric modulator (PAM), CDPPB (3-Cyano-*N*-(1,3-diphenyl-1*H*-pyrazol-5-yl)benzamide; Tocris), to test whether co-administration of CDPPB would block the cognitive-enhancing effects of MTEP. Monkeys were first administered varying doses of CDPPB (0.001, 0.01, and 0.1 mg/kg) to identify a dose that had no effect on its own. This dose was then paired with the cognitive-enhancing dose of MTEP for pharmacological challenge. This protocol was important for ensuring that additive effects of the two agents would not obscure interpretation of the drug effects.

#### Data analyses

The effects of increasing doses of MTEP on working memory performance were analyzed using the Friedman statistic (a non-parametric 1-ANOVA-R) given the non-Gaussian distribution of the data, followed by pairwise comparisons (SPSS, IBM). Challenge of the MTEP response with CDPPB was assessed with a paired *T* test. *P* < 0.05 was predetermined as the threshold for statistical significance.

## Results

### Physiology

**Fig. 2:**
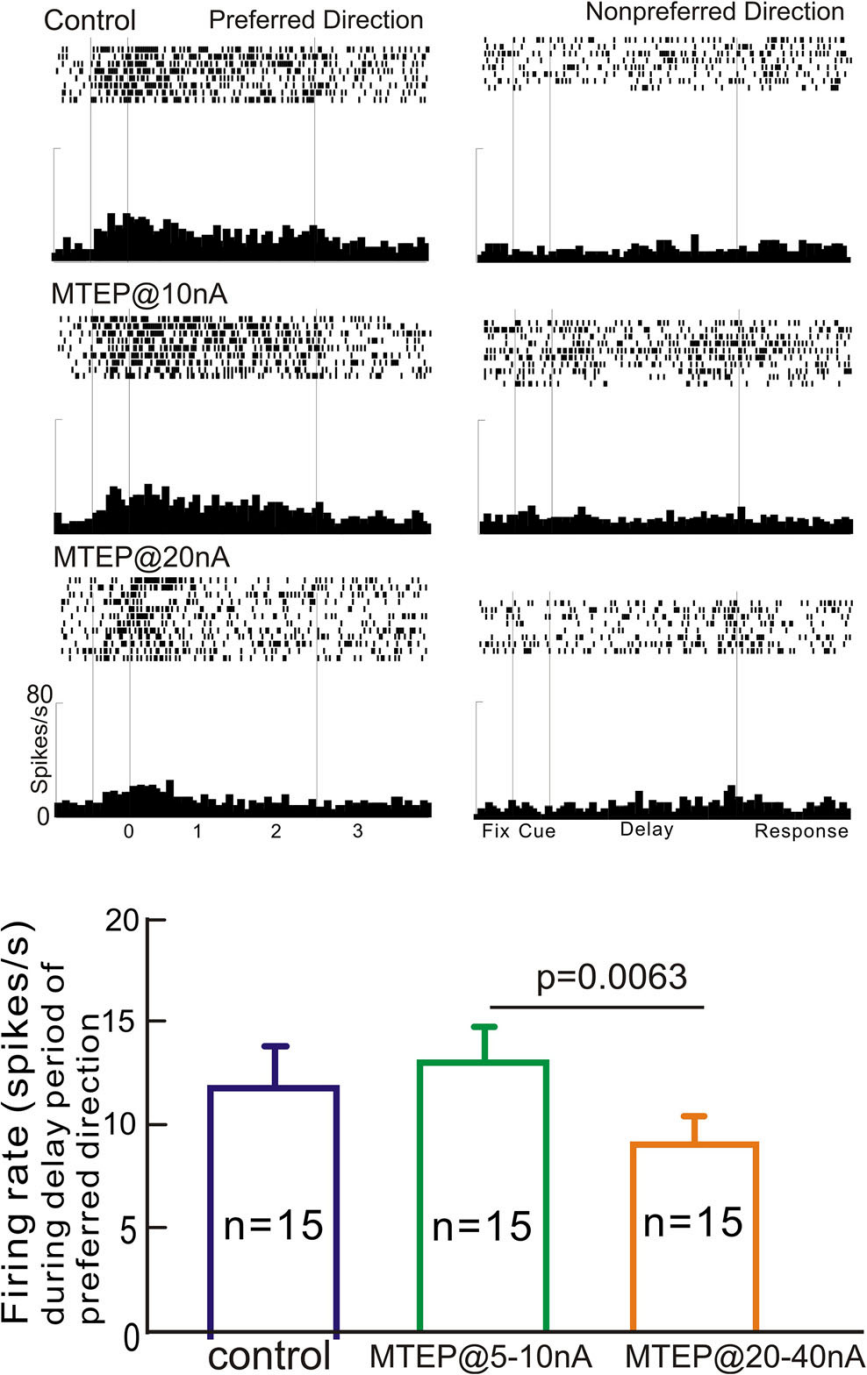
The effects of MTEP on dlPFC Delay cell firing. **a** An example neuron which showed a small increase in firing with iontophoresis of a low dose (10nA), but reduced firing at a higher dose (20nA). **b** The average response of 15 Delay cells to low (5–10nA) vs. high (20–40nA) dose MTEP application

The current study focused on aging monkeys, as the naturally occurring reduction in Delay cell firing in these animals provides an opportunity for pharmacological enhancement (Wang et al. 2011). Iontophoresis of the selective mGluR5 NAM, MTEP, produced an inverted U dose-response on Delay cell firing in the middle-aged and aged monkey performing the ODR task, but with variable enhancement at low doses. An example, Delay cell is shown in Fig. 2a. This neuron showed a small increase in firing during the delay period following low-dose MTEP @10nA, but significantly reduced firing when the dose was raised to 20nA (two-way ANOVA with Dunnett’s multiple comparisons: significant effect of drug F_directionxdrug_(2, 53) = 8.597, *p* = 0.0006; paired comparisons: preferred direction: control vs. MTEP10nA, *p* = 0.592; control vs. MTEP20nA, *p* = 0.0001; non-preferred direction: control vs. MTEP10nA, *p* = 0.3843; control vs. MTEP20nA, *p* = 0.879).

The average response of all Delay cells to MTEP is shown in Fig. 2b. There was a small, non-significant increase in delay firing at low MTEP doses for the neurons’ preferred direction (5–10nA; note that 5nA is the smallest ejection current possible), producing no effect or a small increase in firing in most cells, but a pronounced increase in firing in one neuron. In contrast, higher MTEP doses (20–40nA) reduced firing for the preferred direction in most Delay cells, although one neuron showed increased firing following the 20nA dose (repeated measures one-way ANOVA with Tukey’s multiple comparisons; significant effect of drug F(1.58, 22.11) = 7.888, *p* = 0.0044; paired comparisons: control vs. MTEP10nA, *p* = 0.5125; control vs. MTEP20nA, *p* = 0.0652; MTEP10nA vs. MTEP20nA, *p* = 0.0063). Thus, MTEP generally produced an inverted U dose-response, but results were mixed.

### Cognitive behavior

#### The effects of MTEP on working memory performance

We tested the effects of systemic administration of MTEP across a wide dose range (0.0001–0.1 mg/kg) in a total of 10 aging rhesus monkeys performing a spatial working memory task. As seen with the physiology, MTEP generally produced an inverted U dose/response, although the effects were noisy and not replicable in all animals.

**Fig. 3:**
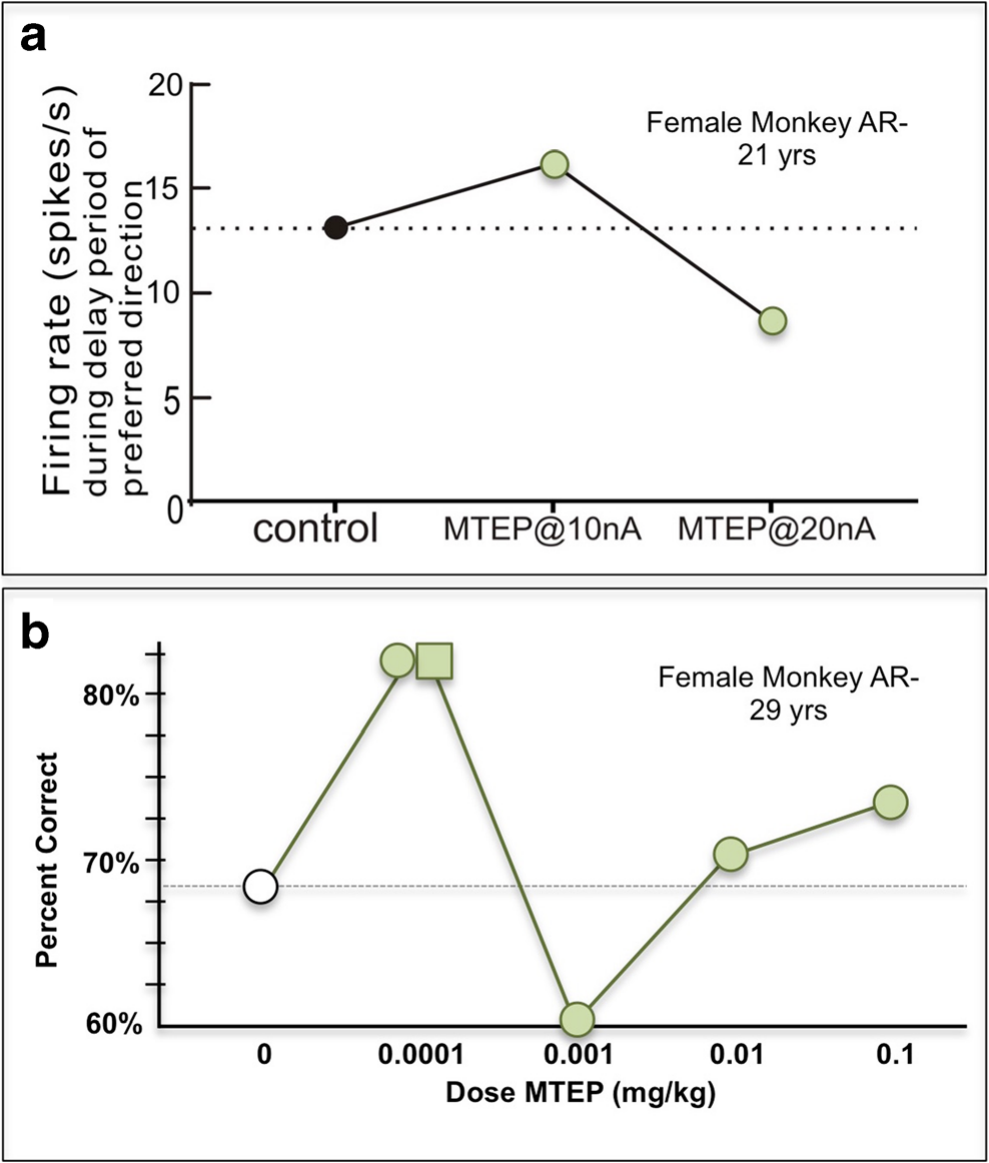
The effects of MTEP on dlPFC neuronal firing (single neuron example) (**a**) and working memory performance (**b**) in aged female monkey, AR. The lowest dose improved neuronal firing and produced a replicable improvement in cognitive performance, while raising the dose reduced firing and performance. Replication indicated by square

We had the rare opportunity to test the effects of systemic MTEP administration in the same aged monkey that had previously participated in the physiology study. This monkey showed replicable improvement at the lowest dose (0.0001 mg/kg), followed by impairment or mixed effects at higher doses (Fig. 3b). These behavioral data are consonant with the effects of MTEP on Delay cell firing in this same monkey, where neurons often showed increased firing at a low dose (10nA), but reduced their firing as the dose was raised (20nA) (Fig. 3a).

**Fig. 4:**
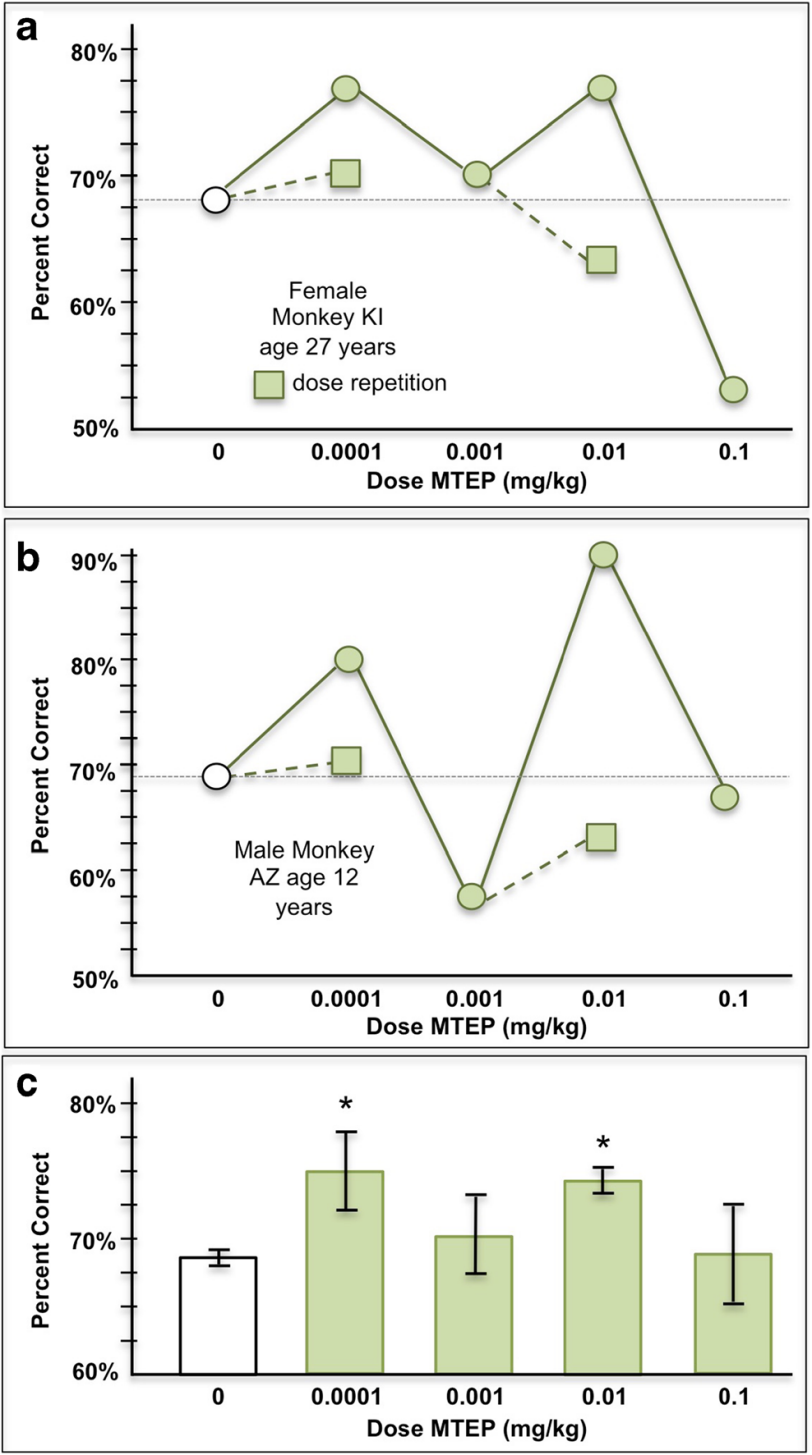
The effects of MTEP on working memory performance in 10 aging rhesus monkeys. **A**, **B** Examples of dose-response curves from two monkeys, showing noisy inverted U relationships, and non-replicable improvements (replications of doses shown as squares). **C** The average (mean ± SEM) response of all to MTEP (0.0001–0.1 mg/kg) administration. There was wide variability in responding, with small but significant improvement following the 0.0001 and 0.01 mg/kg doses, but not the 0.001 or 0.1 mg/kg doses. Significant difference from vehicle at *p < 0.05

MTEP also produced noisy, inverted U dose/response curves in other aged (e.g., Fig 4a) and middle-aged (e.g., Fig. 4b) monkeys. Repetition of enhancing doses failed to replicate in 4 of the 10 monkeys tested (e.g., Fig. 4a, b), while 6 monkeys did show replicable improvement (e.g., Fig. 3b). Overall, the effects of increasing doses of MTEP on spatial working memory performance significantly improved performance, but with an erratic dose-response relationship (Fig. 4c; significant effect of MTEP: Friedman statistic = 13, *p* = 0.0113; paired comparisons were significant for vehicle vs. 0.0001 mg/kg [adjusted *p* value = 0.036 for *n* = 8] and for vehicle vs. 0.01 [adjusted *p* value = 0.029 for *n* = 10]). There were no significant relationships between drug efficacy and the age of the monkeys (all *r* values < 0.25).

#### The effects of MTEP on behavioral ratings

MTEP had little effect on behavioral ratings. Two of the ten monkeys had ratings of ss = − 2 (increased agitation) following the 0.1 mg/kg dose; the remaining animals were unchanged.

#### Blockade with an mGluR5 PAM

**Fig. 5:**
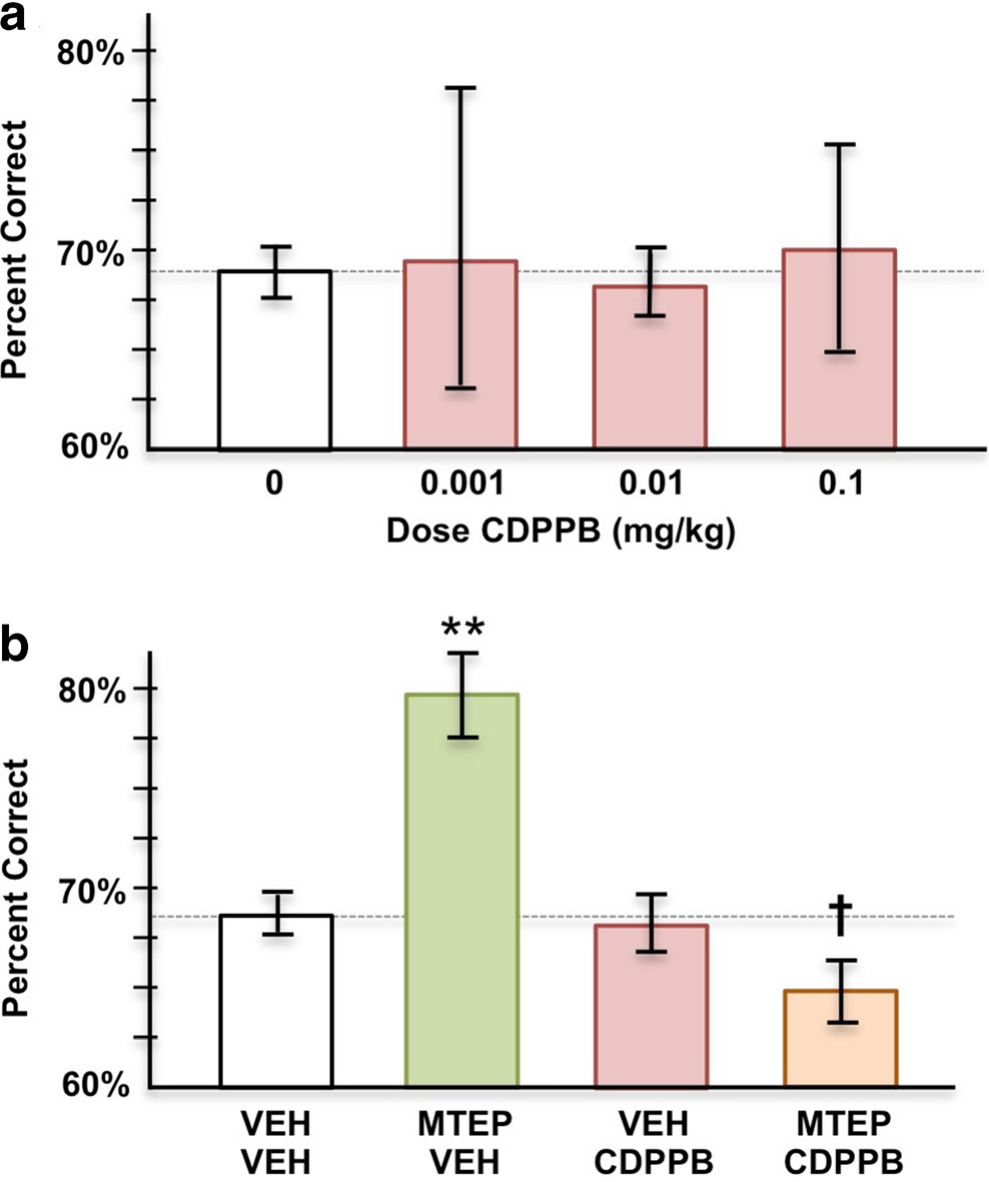
The mGluR5 PAM, CDPPB, reversed the enhancing effects of MTEP in 5 monkeys with replicable improvement. **a** The average (mean ± SEM) response to CDPPB on its own was highly variable. **b** CDPPB co-administration with MTEP blocked the cognitive-enhancing effects of MTEP (*n* = 5). **significantly different from vehicle *p* < 0.01; ^**†**^significantly different from MTEP+vehicle *p* < 0.01

At the end of the MTEP characterization study, the five remaining monkeys that exhibited a replicable beneficial effect with repeated MTEP administration were challenged with the mGluR5 PAM, CDPPB, to test for drug actions at mGluR5. An initial experiment examined the effects of CDPPB alone on spatial working memory performance in these animals to identify an appropriate dosage for challenge of the MTEP enhancing response. Administration of CDPPB (0.001–0.1 mg/kg) by itself had highly variable effects on performance (Fig. 5a). The highest dose of CDPPB that had no effect on its own (0.01–0.1 mg/kg) was then co-administered with an enhancing dose of MTEP in the five monkeys with replicable MTEP improvement. CDPPB co-administration significantly blocked the enhancing effects of MTEP, consistent with actions at mGluR5 (Fig. 5b; Tdep tests: vehicle vs. MTEP, *p* = 0.003; MTEP vs. MTEP+CDPPB, *p* = 0.004). Thus, the enhancing effects of MTEP likely arose from blockade of mGluR5.

## Discussion

Overall, either local or systemic blockade of mGluR5 with MTEP produced a narrow but noisy inverted U dose/response for both dlPFC neuronal firing and spatial working memory performance. Low dose, iontophoretic application of MTEP onto dlPFC Delay cells enhanced task-related firing in some but not all neurons, while slightly higher doses significantly reduced firing in all but one neuron. Systemic administration of MTEP also produced erratic effects on spatial working memory performance, with improvement seen at some, but not all, low doses. Replication of improved performance was only observed in 5/10 monkeys when doses were repeated. However, 5 aged monkeys did show replicable improvement with an identified dose of MTEP, and these enhancing effects were reversed by co-administration of the mGluR5 PAM, CDPPB. As we purposefully selected a dose of CDPBB with no effect on its own, this blockade cannot be attributed to additive drug actions, but rather support mechanistic interactions at mGluR5.

The erratic dose/response curves for working memory performance may arise from the competing effects of MTEP at pre- vs. post-synaptic mGluR5 in the dlPFC (see below), as well as potential opposing actions outside the PFC. For example, higher dose MTEP blockade of mGluR5 in hippocampus might contribute to stronger mnemonic functioning that could partially counteract detrimental actions in dlPFC and lead to overall lack of effect on behavioral performance.

### Potential mechanisms—opposing pre- vs. post-synaptic actions in dlPFC

The physiological recordings show that local blockade of mGluR5 can have opposite effects on Delay cell firing, with a low dose increasing, and a higher dose decreasing, task-related firing. It is likely that actions at pre-vs. post-synaptic receptors contribute to these opposing effects, as summarized in Fig. 1e. Thus, low-dose blockade of post-synaptic mGluR5 may diminish the detrimental effects of feedforward calcium-cAMP-K^+^ actions and increase neuronal firing, while blockade of pre-synaptic mGluR5 would prevail at higher doses, decreasing neuronal firing by reducing glutamate release, e.g., as has been seen in rat medial PFC (mPFC) (Isherwood et al. 2018). The effect of drug may also vary between neurons based on the differing levels of endogenous glutamate actions. For example, the varying levels of enhancement with low-dose MTEP may be related to the divergent levels of endogenous glutamate engagement of post-synaptic mGluR5. Overall, the competing effects of MTEP at pre- vs. post-synaptic mGluR5 may contribute to the erratic dose/response curves seen at the behavioral level.

In addition to “simple” pre- vs. post-synaptic mechanisms on glutamatergic axon terminals vs. spines, rodent research suggests that there may be additional, more complex mechanisms that contribute to MTEP actions. Research on mGluR5 actions in rodent mPFC differs from the current study in several ways: differing species, PFC subregions (mPFC vs. dlPFC), and layers (layer V in rodent mPFC vs. layer III in monkey dlPFC). The subcellular distribution of mGluR5 also differs between primate dlPFC and rodent mPFC, where mGluR5 in primate dlPFC are distributed between dendrites (30%), spines (24%), and axons and axon terminals (17% + 11%) (Muly et al. 2003), but are largely focused on dendrites (72%) in rat mPFC, with very little axonal expression (terminals only 3%) (Fitzgerald et al. 2019). Nonetheless, studies in rodents may be informative regarding potential mechanisms that may extend to primate. For example, rodent studies suggest that the increase in Delay cell firing with MTEP could involve disinhibition of GABAergic signaling. In layer V of rat mPFC, application of an mGluR5 PAM increased IPSCs and reduced neuronal firing, while MTEP increased pyramidal cell firing (Pollard et al. 2014). As mGluR5 are found on a small proportion of GABAergic dendrites in monkey dlPFC (Muly et al. 2003), similar GABA mechanisms may contribute to increased firing with MTEP in primate Delay cells. Conversely, studies in rodents suggest that the reductions in firing with MTEP can involve mGluR5 interactions with endocannabanoid signaling, as dendritic mGluR5 are often localized near synapses with pre-synaptic CB1 receptors (Fitzgerald et al. 2019). In these experiments, mGluR5 stimulation increased the excitation of pyramidal cells through heterosynaptic CB1 receptor interactions that reduced GABA release onto the pyramidal cell dendrite (Kiritoshi et al. 2013). It is not known if these interactions occur in primate dlPFC, but if so, they could contribute to reduced Delay cell firing with mGluR5 blockade.

The large species differences in subcellular localization of mGluR5 caution that results in rodents may not always translate to primates. In particular, the finding that mGluR5 are largely post-synaptic in the rodent hippocampus (Shigemoto et al. 1997) and mPFC (Fitzgerald et al. 2019) suggests that mGluR5 agents may have more consistent effects in rodents than in primates, where the subcellular distribution of mGluR5 in the cortex is both more expansive and more complex (Muly et al. 2003). For example, in vitro studies of slices from rat brain show that post-synaptic mGluR5 stimulation can induce persistent firing of layer V neurons in medial PFC (Sidiropoulou et al. 2009) and anterior cingulate cortex (Zhang and Séguéla 2010) and in layer III neurons in entorhinal cortex (Yoshida et al. 2008). As these are studies of cortical slices where circuit inputs are usually interrupted, drug effects likely arise from inherent properties of the neurons, rather than from recurrent excitation from local circuits, e.g., from internal calcium release (Zhang and Séguéla 2010). These beneficial effects of mGluR5 on neuronal excitability may also occur in primates, and may be blocked by MTEP, contributing to the noisier dose/response.

### Clinical relevance

#### mGluR5 in human brain

In vivo PET imaging of mGluR5 with [(11)C]ABP688 shows that the dlPFC is one of the areas of densest mGluR5 expression in human brain (DuBois et al. 2016). In contrast, primary sensory cortices have lower levels of mGluR5 expression, consistent with the fundamental differences in molecular regulation between association cortices and primary sensory cortices in primates (Yang et al. 2018). These PET imaging data suggest that mGluR5 pharmaceutical agents would have prevalent actions in human dlPFC, and that the mixed effects seen in the present study may also be relevant to human drug trials assessing cognitive function.

#### Fragile X syndrome

Research in mouse models of Fragile X syndrome has found that genetic or pharmacological reduction in mGluR5 signaling reduced abnormal behaviors in mice (Bear et al. 2008; Bear et al. 2004). For example, an mGluR5 NAM reduced repetitive behaviors and restored social behaviors such as interactive sniffing (Silverman et al. 2012). However, clinical trials of mGluR5 NAMs in adolescents and adults with Fragile X syndrome have failed to ameliorate behavioral symptoms (Berry-Kravis et al. 2016; Berry-Kravis et al. 2018). It may be that more targeted measures are needed to assess drug efficacy, especially to capture measures of social cognition that rely heavily on visual stimuli in primates, and often involve expansive PFC circuits (Wittmann et al. 2018). In particular, a recent study of the mGluR5 NAM, mavoglurant, found positive results on a test of social gaze in adolescents and adults with Fragile X syndrome (Hessl et al. 2019), suggesting that a more targeted approach on social cognition may be more sensitive and fruitful. Interestingly, mavoglurant was effective at low, but not high, doses (Hessl et al. 2019), reminiscent of the inverted U dose-response seen in the current study. The monkey data also suggest that there may be subgroups of individuals who show greater benefit, and that experimental designs could benefit from analyses of subgroup responding, as well as the inclusion of lower doses,. However, the monkey data also caution that a narrow inverted U dose-response, and individual variation in best dose, may make it challenging to find correct dosing for human subjects. Thus, these studies may need to perform individual dose titration to identify optimal parameters for each patient.

#### Aging and AD

mGluR5 blockade has also been suggested as a therapeutic strategy for AD. There is some evidence that mGluR5 are reduced with advancing age in rat PFC, and that this contributes to working memory impairment, as infusion of MTEP into the mPFC in young rats produced a small impairment in performance (Hernandez et al. 2018). mGluR5 levels may also reduce with age in human brain (DuBois et al. 2016), but the brain imaging data indicates that this is a function of age-related decreases in gray matter, e.g., loss of synapses (DuBois et al. 2016). mGluR5 have been implicated in the toxic effects of amyloid Aβ pathology in AD, where mGluR5 stimulation can increase the expression of the amyloid precursor protein APP (Westmark and Malter 2007), and Aβ oligomers engage the mGluR5-PrP complex to initiate toxic signaling events (Hamilton et al. 2015; Renner et al. 2010; Um et al. 2013). mGluR5 antagonists have been proposed as a treatment for AD (Kumar et al. 2015; Sokol et al. 2011), largely based on research from rodent models, e.g., where knockout of mGluR5 (Hamilton et al. 2014) or negative allosteric modulation of mGluR5 (Abd-Elrahman et al. 2020) can reduce amyloid pathology and protect cognition. However, the inconsistent and sometimes detrimental effects of MTEP on spatial working memory performance in the current monkey study suggest that blockade of the glutamate site on mGluR5 may not be helpful for treating AD. Blocking pre-synaptic mGluR5 in dlPFC may reduce glutamate release and reduce neuronal firing, and thus counteract any helpful effects of post-synaptic blockade. More selective targeting of the Aβ site on the mGluR5-PrP complex may be a more fruitful strategy (Haas et al. 2017), avoiding interference with the beneficial effects of endogenous mGluR5 stimulation.
